# Client and Pantry Factors Influencing Transportation-Related Barriers Among Users of Food Pantries: A Cross-Sectional Analysis

**DOI:** 10.3390/foods14213673

**Published:** 2025-10-28

**Authors:** Jackson F. Stone, John R. Bales, Jonathan D. Harris, Claire E. Harper, Joshua J. Scott, Joseph J. Kotva, David S. Lassen

**Affiliations:** Indiana University School of Medicine, South Bend, IN 46617, USA

**Keywords:** food insecurity, transportation, food bank, food pantry, barriers

## Abstract

Food insecurity is a pervasive public health issue in the United States. While food pantries attempt to alleviate this issue, their effectiveness is limited by structural and logistical barriers that affect service accessibility. Transportation is a frequently underexamined barrier for individuals trying to access food aid. The purpose of this study is to assess the interplay of client- and pantry-level characteristics and their influence on food aid accessibility across several transportation modalities. This cross-sectional survey study collected data from 430 food pantry clients concerning their demographics, transportation methods, and perceptions of transportation barriers. Pantry characteristics were also collected focusing on transportation infrastructure and operational policies. Individual and grouped comparisons were made between transportation methods in relation to pantry visitation, with those walking, biking, and taking a bus to the pantry grouped to compare to those taking a car. Higher food insecurity score, smaller household size, single relationship status, and race were independently associated with increased odds of walking, biking, or taking a bus to the pantry. Having closer bus stops, more bus lines, and no monthly use limits were independently associated with increased odds of walking, biking, or taking a bus to the pantry. Several characteristics were associated with specific transportation modalities when accessing food aid. Our results are particularly concerning given the increased food insecurity and additional vulnerabilities seen in those who walk, bike, or take the bus to the pantry. Transportation disadvantage may be ameliorated by less restrictive pantry use policies and more robust public transit.

## 1. Introduction

Food insecurity—limited or uncertain access to sufficient, safe, and nutritious food, or inability to obtain food in socially acceptable and dignified ways—is a pervasive public health issue in the United States [1]. In 2023, the U.S. Department of Agriculture estimated that 13.5% of American households experienced food insecurity at some point during the year, an increase from 12.8% in 2022 [2]. Food insecurity is associated with a range of physical and mental health concerns, including increased risk of chronic diseases, depression, and disordered eating behaviors [3,4,5,6,7,8,9]. One primary mechanism to alleviate food insecurity is the food aid system, which includes a network of food banks, food pantries, and community-based distribution programs. Among these, food pantries play a distinct role by offering direct, community-based food access to individuals [10,11]. While food pantries serve as critical access points, their effectiveness is shaped by structural and logistical factors that influence who is able to access their services and how frequently. Amidst these considerations, transportation remains a frequently overlooked yet pivotal factor influencing an individual’s ability to access food aid [12].

While access to reliable transportation is routinely cited as a limiting factor for Americans attempting to access grocery stores [13,14,15], there is limited research on how transportation shapes access to food aid. Interview studies have highlighted transportation as a significant problem for food pantry access, with clients citing barriers including cost, time, transit schedules, and ability to transport goods. Many added that these factors may influence what method of transportation they take to the pantry [16,17,18]. Yet, the current literature fails to quantify how and why individuals alter their transportation methods when accessing food pantries. The ability to make such adjustments suggests a level of flexibility in transportation access, which is unlikely to be shared by all clients.

For many, limited transportation may directly translate to limited access to food. In the context of grocery access, previous work has demonstrated that those with limited transportation are more likely to be food insecure compared to those with access to a car [13,19,20]. While these findings highlight a relationship between transportation and food access, to our knowledge no studies have examined how transportation resources affect food insecurity rates among recipients of food aid.

To more fully understand the interplay between transportation and food access, it is important to recognize the people it affects. Disparities in food security have consistently been shown to disproportionately affect minority populations, specifically African American and Hispanic adults [21,22]. Yet, the current literature rarely attempts to connect food insecurity with transportation resources. In a systematic review identifying higher rates of food insecurity among minority households, only one of ninety-eight reviewed articles attempted to investigate transportation as a contributor [21]. This oversight persists despite a frequent association between transportation disparities and minority populations [23,24,25]. There is a similar paucity of research attempting to characterize how other demographic variables relate to transportation and food aid access [15,26,27]. However, the complexities of this topic extend beyond individual-level characteristics.

A discussion of transportation resources and food aid accessibility necessitates consideration of food pantry policies and the built environment. Previous interview studies of food pantry clients have highlighted the restrictive nature of food pantry operating times, among other policies, especially for those without access to a car [16,18,28]. For many, the ability to access food relies on the alignment of client, pantry, and transit schedules [29,30]. Moreover, the accessibility of a food pantry for transit-reliant clients is dependent most simply upon the existence of transit lines and stops near food aid organizations. When a bus is available, clients report taking only the food items they can carry, suggesting the ability for clients to choose their items may facilitate pantry use among transit users [16,31]. The intersection of infrastructure and food access also extends to walking and biking. Previous work has associated walkability and presence of pedestrian infrastructure, such as sidewalks and bike lanes, with better food access [32,33,34,35,36]. Currently available literature underlines the complexity of food access as it relates to individual city- and pantry-level factors, but few attempt to measure the interplay between these variables in relation to food pantry utilization [16,18,28,29,30,31,32,33,34,35,36].

This is a quantitative, cross-sectional study that draws on a large cohort of food aid recipients and combines client- and pantry-level characteristics to address gaps in the literature and clarify how transportation resources shape access to food aid. The study sought to assess discrepancies in transportation modality when accessing food aid and whether these have any effect on food security. We hypothesized heightened disadvantage for those walking, biking, or taking a bus to the pantry and thus looked to explore whether perceptions and demographic characteristics differed by group. Further, this study aimed to assess if pantry policies—such as limits on use or client choice—or transportation infrastructure—such as number of bus lines or the presence of sidewalks—are associated with the use of specific types of transportation to access pantries. Taken together, this study will help inform targeted strategies for facilitating equitable access to food aid.

## 2. Methods

### 2.1. Study Design and Recruitment

Food aid services across the greater St. Joseph and Elkhart County areas in Northern Indiana were contacted by email or phone for potential participation. The initial contact email or phone conversation discussed study rationale and detailed study procedures. Ten services agreed to let study staff administer surveys at their location before the deadline of July 2023. Based on pantry schedules, dedicated dates of survey administration were identified at each respective location. At least one study staff member was present for survey administration.

This was a prospective, cross-sectional study among individuals accessing food aid in the greater St. Joseph County area with survey administration occurring between June and July 2023. Potential subjects were approached for research participation while waiting in line at food pantry locations. Eligible subjects had to be at least 18 years of age, seeking services at the participating food pantry, and able to read or speak in English or Spanish. Prior to survey administration, a study information sheet was provided, and trained study staff explained study procedures and data utilization to potential study participants through a verbal script. To avoid collecting identifying information, study participants indicated informed consent via a checkbox prior to survey administration. A five-dollar gift card to Walmart was provided to the 474 patrons who completed the survey.

### 2.2. Measures

#### 2.2.1. Survey Questions

As part of a large analysis to evaluate barriers to food aid acquisition and utilization, a 22-question survey was developed and administered. Select questions applicable to our research objectives were used for data analysis and are described below. Eligible and consenting participants were asked to provide the following demographic information: age, race/ethnicity, relationship status, household size, and education level. Participants were then asked to answer questions concerning food insecurity and perceptions surrounding accessing food aid. Food insecurity questions were collected from the Food Insecurity Experience Scale, as previously described by Cafiero et al. and Sheikomar et al. [37,38]. The scale asks the study participant eight binary questions regarding whether they have experienced unique food insecurity situations at least once in the past 12 months. One point is awarded for each scenario experienced, with larger scores corresponding to worse food insecurity. Three unique perception questions were also utilized. The first asks participants to use a four-point Likert scale to answer, “How much does each of the following affect your decisions when selecting food at this pantry?” in regard to “Ease of transport” with options ranging from “large effect” to “no effect.” The final two perception questions asked participants “How much do you agree with the following statements” with the statements being “Travel is frequently difficult for me” and “Travel is frequently a financial burden for me.” Participants responded using a five-point Likert scale from “strongly disagree” to “strongly agree.” Finally, participants were asked to report both how they arrived to the pantry on the day of survey and the method of transportation they use most frequently, from the following options: “drive a personal vehicle,” “get a ride in a friend’s vehicle,” “ride a bicycle,” “take the bus,” “use a rideshare service such as Uber or Lyft,” or “walk.”

#### 2.2.2. Pantry-Level Characteristics

Pantry characteristics, including days of operation, use limits, provision of ready-made meals, and client choice were collected from a combination of pantry websites, discussions with staff, and direct observation. Days of operation were categorized in a binary fashion as either open less than three days per week or open three days per week or more. Use limits were binarized on whether a patron was allowed to access a pantry more than once per month. This classification was based on the restriction guidelines of many local sources of food aid [39,40]. Provision of ready-made meals was binarized on whether the establishment utilized a kitchen component or not. Finally, there was variability in the level of choice clients had when selecting food at each location. This ranged from no choice, such as fully pre-packaged boxes of items, to full choice, such as menu selection or an individual shopping experience. Partial choice was a combination of the two.

The presence of sidewalks, bike lanes, and bus lines was collected utilizing direct observation, 2023 Apple Maps and Google Maps data, and 2023 South Bend Transpo route maps. Sidewalks were binarized as present or absent, with presence defined as a paved walking path dedicated to pedestrian traffic that extends from, or is adjacent to, the pantry entrance and then branches out at two or more intersections. Bike lanes were also binarized as present or absent, with presence defined as a dedicated bike lane or “multi-use path” as established by the Michiana Area Council of Governments (MACOG) within a 0.5-mile walking radius. This distance was chosen as a conservative estimate of how far individuals are willing to walk for food [41]. The number of bus lines were counted for each pantry based on how many lines had a stop present within a 0.5-mile walking radius [41]. For each pantry location, the distance to the nearest bus stop was estimated using the average between satellite imaging service estimates.

### 2.3. Statistical Analysis

Appropriate sample size was calculated based on an estimated food insecure population in St. Joseph County of about 42,500 [42]. To our knowledge, no previous literature attempts to determine the percentage of those walking, biking, and taking a bus when accessing food aid among this niche population. Thus, we used a conservative population proportion of 20% [13,43], the estimated prevalence of those walking, biking, or taking a bus when obtaining food aid. With the given population size, expected prevalence, Z-score of 1.96, and power of 80%, a priori power analysis revealed a sample size of at least 245 individuals to ensure our target population is being adequately represented.

All statistical analysis was completed using R.Studio (version 2023.12.1). Of the 474 individuals who agreed to participate, 430 indicated their method of transportation to the pantry on the day of surveying and thus were included in our analyses. Not every participant answered all the questions listed in the survey. Because there are effectively different sample sizes for many of these questions, relative sample sizes for each answered question are listed in the results as appropriate. For binary analysis of transportation groups, we combined those walking, biking and taking a bus to compare to those who arrived to the pantry by personal vehicle, friend’s vehicle, or rideshare service. Results were suspected to be similar across walkers, bikers, and those taking the bus as robust public transportation is often indicative of a more walkable setting and these modalities are similarly limited in their reach compared to a car [36,44]. Descriptive statistics were reported using means and standard deviations for continuous variables, and frequencies and percentages for categorical variables as needed. Nonparametric tests were utilized under the assumption of non-Gaussian distributed data as determined by quantile-quantile plots and Shapiro–Wilk normality tests. Differences between continuous variables were assessed using two-sided Mann–Whitney U tests for all comparisons. Chi-squared analysis was used for comparison between categorical variables. Kruskal–Wallis tests were utilized to compare household size and food insecurity scores when separating transportation methods across all transportation modalities. Further multivariable logistic regression analysis was undertaken in two instances: (1) to assess the association between food insecurity and transportation type when accounting for demographic data, (2) to assess the association between pantry-level characteristics and transportation type. In the former, only demographics with statistically significant differences between transportation types were included in the adjusted model. The latter analysis included number of bus lines, distance to bus stop, days of operation, use limits, and presence of sidewalks as independent variables and transportation type as the dependent variable. High multicollinearity was anticipated between independent variables within the second analysis. Thus, variance inflation factors and Lasso regression were used to inform variable selection. Provision of ready-made meals and presence of bike lanes were removed; distance to nearest bus stop and number of bus lines were separated. Results were reported using odds ratios with 95% confidence intervals. A *p*-value of 0.05 was set as a threshold of clinical significance across all comparisons.

## 3. Results

### 3.1. Transportation

#### 3.1.1. Changes in Transportation

Of the 430 survey participants who disclosed their mode of transportation to the pantry on the day of the survey, 358 arrived by personal vehicle, a friend’s vehicle, or ride-share services, while the remaining 72 arrived by walking, biking, or taking a bus. Comparing how patrons most frequently traveled in daily life to how they arrived at the pantry revealed a statistically significant shift; a large portion of those who would normally walk, bike, or take a bus are instead taking a car to the pantry (Table 1).

#### 3.1.2. Transportation and Food Insecurity

When stratified by transportation type, the Food Insecurity Experience Scale failed to meet statistical significance (*p* = 0.060) [37,38]. Small sample sizes in our stratification likely contributed. Notably, those arriving to the pantry via a personal vehicle had the lowest average food insecurity scores of all transportation types (Table 2). When grouped together, food insecurity scores for those walking, biking or taking a bus were higher than those arriving by car (FI Score: WBB 5.90 vs. Car 5.02, *p* = 0.037).

#### 3.1.3. Client Perceptions on Transportation

Those who walk, bike, or take a bus to the pantry more often reported that ease of transportation plays at least a moderate effect in food item selection (*n* = 406, WBB 52% vs. Car 33%, *p* = 0.0056) (Table 3). Additionally, although not statistically significant, those who walk, bike, or take a bus to the pantry agreed that transportation is difficult for them when compared to the car cohort (*n* = 278, WBB 63% vs. Car 49%, *p* = 0.0867). There were no differences between client perceptions on whether transportation is a financial burden between cohorts (*n* = 318, WBB 61% vs. Car 60%, *p* = 0.9162).

### 3.2. Client Characteristics

#### 3.2.1. Demographics

The sociodemographic characteristics of the 430 study participants are represented in Table 4. Participants had a mean age of 54.04 years (SD ± 14.73). Most participants had completed High School, a GED or greater. The majority of participants were more often single, never married, and identified as white. Mean household size of participants was 3.20 persons (SD ± 1.91).

In an analysis of those accessing food aid by car versus those walking, biking, or taking a bus to the pantry, client-reported ages were on average older for those arriving by car. Client-reported relationship status varied with statistical significance among the transportation groups. A binarized sub-analysis demonstrated that there was a statistically greater proportion of non-partnered patrons walking, biking, or taking a bus to the pantry. Client-reported education did not statistically vary among transportation modalities. Additionally, client-reported race and/or ethnicity revealed statistically significant differences between the two groups (Table 5). In a sub-analysis, it was identified that those walking, biking, or taking a bus to the pantry were more likely to identify as belonging to a minority population (WBB 60% vs. Car 38%, *p* = 0.001).

#### 3.2.2. Household Size

When participants were asked to report the number of people living in their household, including themselves, household size differed significantly across modes of transportation (Table 6). A more specific analysis revealed that those taking a car to the food pantry reported a larger mean household size than for those walking, biking, or taking a bus (WBB 2.34 vs. Car 3.33 persons, *p* = 0.007).

#### 3.2.3. Logistic Regression of Client Characteristics

A multivariable logistic regression examined demographic predictors of walking, biking, or taking the bus to the food pantry (Table 7). Higher food insecurity scores were associated with increased odds of using these transportation modes (OR = 1.28, CI = 1.02–1.67), while larger household size was associated with lower odds (OR = 0.53, CI = 0.32–0.78). Relationship status was significant as well: compared to single, never-married patrons, those who were divorced (OR = 0.18, CI = 0.04–0.70) or married (OR = 0.09, CI = 0.00–0.62) had reduced odds of walking, biking, or taking the bus. Race was also a significant factor, with African American patrons more likely to walk, bike, or take the bus when compared to white patrons (OR = 4.48, CI = 1.21–17.45), as were patrons identifying with less frequently represented racial groups in the “Other” category, including Asian, American Indian/Alaskan Native, and Pacific Islander identities (OR = 10.14, CI = 1.22–90.16).

### 3.3. Pantry Characteristics

#### 3.3.1. Operations

An assessment of how frequently food aid organizations are open to clients reveals that those walking, biking, or taking a bus to the pantry were more likely to be at a pantry that is open three or more days a week (Table 8). Similarly, those walking, biking, or taking a bus to the pantry were more likely to be at a pantry that allowed patrons to get food more than once a month.

#### 3.3.2. Food and Distribution

Our analysis reveals that organizations serving ready-made meals had a greater proportion of those accessing the establishment by walking, biking, or taking a bus. However, when comparing transportation groups across the levels of client choice offered by pantries, no statistically significant difference was observed (Table 9).

#### 3.3.3. Transportation Infrastructure

Components of transit and pedestrian infrastructure were assessed in relation to clients’ mode of transportation. Regarding distance to the nearest bus stop and number of bus lines, statistically significant differences were observed across the two transportation groups. The relative frequency of those walking, biking, or taking a bus to the pantry was lowest at the establishments with the furthest bus stops and the fewest available bus lines. Further, the presence of sidewalks and the presence of bike lanes were each associated with significantly greater proportions of those walking, biking, and taking a bus to the pantry (Table 10).

#### 3.3.4. Regression Analysis of Pantry Characteristics

Two multivariable logistic regressions examined pantry characteristics associated with patrons accessing the pantry by walking, biking, or taking the bus compared to using a car (Table 11a,b). Due to high collinearity between distance to nearest bus stop and number of bus lines, these variables were utilized in separate regressions. In their respective regressions, distance to the nearest bus line and number of bus lines were strong predictors of walking, biking, or taking the bus to the pantry. Each also demonstrated a stepwise increase in odds as distance to bus stop decreases or number of bus lines increases. In both analyses, pantries restricting use to one visit per month were associated with decreased odds of walking, biking, or taking the bus. The days of operation and the presence of sidewalks were not significantly associated with transportation method.

## 4. Discussion

This was a survey of food aid recipients with a focus on transportation disparities, collecting data on both client demographics and pantry characteristics to examine associations with transportation modality and identify barriers to equitable food access. We found there was a significant shift towards car use when accessing food pantries, and that on average those walking, biking, or taking a bus to the food pantry were more food insecure than those arriving by car. This group of walkers, bikers, and bus users was more likely to cite transportation as a limiting factor in food selection. They were also more likely to be single, identify as members of racial or ethnic minority groups, and have smaller household sizes. Pantries with more bus lines, closer bus stops, and decreased limits on use seemed to be favored by those walking, biking, and taking the bus. These findings expand on previous work emphasizing the importance of geographic accessibility and intentional pantry policies in supporting underserved populations [45,46,47]. Overall, this study demonstrated that transportation remains an under-addressed barrier in the context of food aid, with many pantries insufficiently accessible to individuals without access to a car—a population who faces compounding vulnerabilities.

### 4.1. Transportation Methods, Food Insecurity, and Perceptions

Our preliminary analysis of transportation methods confirms our initial hypothesis that transportation resources are an important component of food aid accessibility, a finding that aligns with current literature [13,20]. Furthermore, the apparent increase in car travel and reduction in those walking, biking, and taking the bus to the pantry suggested that pantry access may depend on, or be significantly facilitated by, access to a private vehicle. These findings justify a focused analysis on patrons with and without car access. Specifically, it was examined whether those without access to a car face measurable disadvantages, how they perceive these challenges, and how pantry- or city-level characteristics may reinforce or alleviate disparities.

With an observed shift towards car utilization when accessing food aid, it was hypothesized that those without access to a car would be disadvantaged. Specifically, this group was anticipated to report heightened food insecurity. The results indicated individuals who walked, biked, or took the bus to reach the pantry had significantly higher food insecurity scores than the car group. Additionally, when adjusting for confounding variables, having a higher food insecurity score was associated with increased odds of walking, biking, or taking the bus. Together, these findings suggested a compounding disadvantage, wherein those facing more severe food insecurity also encounter greater transportation-related barriers to accessing relief. This relationship reinforces previous findings in the literature, where individuals most in need of food aid may also be the least equipped to consistently and conveniently access it [13,48].

To further assess disadvantages among patrons walking, biking, or taking a bus, participants were surveyed to evaluate perspectives on transportation-related burdens. Our findings suggest that transportation goes as far as affecting food selection, as transportability was indicated to be a more important consideration for those who walked, biked, and took a bus when selecting food. This likely reflects the physical constraints of carrying groceries. This constraint has been identified in previous interviews with food pantry patrons; however, these studies did not attempt to quantify this across a larger cohort [49,50]. Additionally, our analysis highlights the perceived general difficulty of those accessing a pantry, with stratified analysis revealing meaningful differences by transportation type. Patrons with personal vehicles likely face less difficulty than those relying on a friend’s car or rideshare services, highlighting the nuance lost in binary categorizations of car access. Walkers, bikers, and public transit users face longer travel times and limited transit routes, coupled with economic constraints which exacerbate challenges for low-income households [51]. Interestingly, while perceived transportation difficulty varied, perceptions of financial burden did not differ significantly between groups, likely reflecting broader economic stressors common to all pantry clientele.

### 4.2. Client Characteristics

While an analysis of demographic factors is warranted to ensure generalizability and investigate possible confounders, we also believe it is important to characterize underserved populations that future work may be attempting to reach. We were specifically interested in elucidating the connection between household size and transportation as it relates to food security, an understudied topic in the current literature. It was discovered that those accessing the pantry by car had on average more total persons living in their household. Additionally, those living in smaller households were more likely to walk, bike, or take the bus when adjusting for demographic variables. These findings are possibly due to a variety of factors including the probability of car ownership per house increasing as the number of adults increases, the potential for group-funding of a vehicle, and the necessity of car transport for bulk food aid when providing for larger household sizes [52]. Furthermore, those in smaller households may have less social support, aligning with prior work linking social isolation to higher food insecurity [53,54,55]. Thus, household size does not merely reflect a demographic trait but may also serve as a proxy for broader relational vulnerabilities that may impact how individuals access food aid. This aligns with our analysis of relationship status which demonstrated a disproportionate number of single patrons accessing food aid regardless of transportation type [56]. Non-partnered patrons also represent a larger proportion of those walking, biking, or taking a bus to the pantry than those arriving by car. Partnership status may relate to household size as there is an increased chance for cohabitation and children in a partnered relationship; however, our regression indicates that household size is associated with car use independent of relationship status [57]. Furthermore, our regression revealed individuals currently married or divorced are less likely to walk, bike, or take the bus to access food aid when compared to single, never-married individuals, suggesting a possible conferred advantage in transportation access within a food aid context. While existing literature rarely examines the intersection of partnership status and transportation modality [58], divorce has been demonstrated to be associated with economic disadvantage and decreased car ownership [59,60]. However, our findings indicate that divorced food aid patrons were more likely to access the pantry by car. The potential reasons for this result are likely complex and may indicate that our subpopulation of divorced patrons accessing food aid may be different from the general divorced population.

Our discussion of demographic associations with transportation would be incomplete without consideration of race and ethnicity. Minority populations consistently represent a disproportionate share of the food-insecure population in the United States, and this pattern is shaped by intersecting socioeconomic barriers, structural racism, and historical disinvestment in marginalized communities [61,62,63,64]. While our study population aligns with this national trend, clients arriving to the pantry by walking, biking, or taking the bus were more likely to identify with a racial or ethnic minority group compared to those arriving by car. These findings are consistent with prior studies documenting disparities in vehicle access and car ownership across racial and ethnic lines [24,25].

When controlling for other demographic characteristics, race remained a significant predictor of transportation method. Specifically, patrons identifying as African American had significantly higher odds of walking, biking, or taking the bus to access food aid. This pattern is reinforced by previous research demonstrating that Black households are more likely to be carless and rely on public transportation or non-motorized forms of mobility, a trend not solely attributable to income or urban location [23,24,25].

Additionally, our regression analysis revealed that clients who were grouped as Other, of whom more than half identified as American Indian/Alaskan Native, were also significantly more likely to arrive by walking, biking, or taking the bus. Existing evidence suggests that these communities often experience systemic geographic isolation and infrastructural underinvestment, leading to a reliance on private transportation frequently in the form of carpooling [65,66,67]. However, there is limited literature regarding resource disparities outside of reservation settings. It is likely that many inequities still exist for Native Americans in this context, and our results suggest that access to private transportation is limited for this sub-group.

Our analysis did not find statistically significant associations between walking, biking, and taking the bus and identifying as Hispanic or multiracial. This may reflect the distinct transportation strategies employed within the Hispanic community. Previous research has shown that Hispanic households, particularly immigrant families, frequently rely on shared transportation methods such as carpooling or family vehicle sharing [68,69]. Thus, the lack of significant differences in walking, biking, or bus use among Hispanic clients is consistent with these established patterns of car access and resource-sharing networks [69,70].

While our analysis indicates that minority populations may disproportionately walk, bike, or take the bus to access food aid, it is important to recognize that historically, minority neighborhoods often have less access to public transportation, and worse overall investment into transportation infrastructure [71,72]. This only compounds the difficulty these groups may face when attempting to access food aid and may indicate an opportunity for a targeted approach in improving transportation infrastructure.

### 4.3. Pantry Characteristics

Our analysis of pantry characteristics reveals how organizational features may influence the transportation patterns of food pantry clients. These findings offer critical insight into the infrastructure and policies that either enable or constrain access for transportation-vulnerable populations.

Across several measures, one key factor identified was pantry access policy. Our findings suggest that pantries that are open more frequently throughout the week and place less limitations on use are preferred by those who walk, bike, or take the bus. However, only monthly use limits remained significant in our multivariable analyses. This may suggest that increased temporal flexibility can compensate for the rigidities of transit-based travel. Such constraints include cost and fixed transit schedules [73,74]. Less restrictive use limits may also alleviate the need for bulk transportation for those who walk, bike, or take the bus. It is important to note that limits on pantry use may be imposed for a variety of reasons, some of which are limited food supply, volunteering capacity, and the imperative to distribute resources equitably across a high-need population [28,49,75]. This is made further complex by a combination of regulations from federal, state, and local governments, as well as agreements with suppliers and food banks [76,77,78]. Therefore, monthly use limits often reflect not only rationing practices but also broader structural limitations that shape how and when clients can access food aid.

Pantry food distribution format also appears to shape access. Specifically, pantries offering ready-made meals attracted a higher proportion of clients walking, biking, and taking the bus. This may speak to the practical needs of many in this population, such as housing instability or limited capacity to cook or store food. However, interpretation of this result warrants caution as it was excluded from the regression due to high multicollinearity. Interestingly, the ability for clients to shop for specific items had no effect on transportation method. It was anticipated that those who walk, bike or take the bus to the pantry would favor complete or partial choice in selecting food items due to the level of flexibility it grants patrons to take what they can comfortably transport [16]. While the majority of those walking, biking, and taking the bus did access complete and partial choice pantries, the same is true for those in the car group. These findings may suggest that principles of food dignity and autonomy are favored by all and may not directly influence transportation-related access. However, these findings could also be explained by a disproportionate representation of choice-model pantries in our area, or possibly a greater shift towards client-choice models for food aid organizations in general [79,80,81].

Beyond pantry-specific policies, local infrastructure plays a critical role in food aid accessibility. Our initial analysis of pedestrian infrastructure suggests that pantries with sidewalks and bike lanes are more attractive to those walking, biking, or taking a bus to the pantry. However, the presence of sidewalks was an insignificant predictor in our regression, and the presence of bike lanes had to be excluded due to high multicollinearity. With respect to transit infrastructure, pantries with closer bus stops were significantly favored by those walking, biking, or taking the bus. Our findings support prior research linking spatial proximity to food-related service utilization among carless households [82,83]. Similarly, pantries with a greater number of bus lines within a half-mile walking radius were preferred by those walking, biking, or taking the bus. This may reflect how a greater number of nearby bus lines potentially reduces route complexity and minimizes transfer requirements, making the pantry more accessible from multiple neighborhoods. Together, these results emphasize the importance of mitigating travel burdens for clients navigating a limited transit system. Of note, the number of bus lines and closeness of bus stops were found to be highly collinear. While this is likely due to the increased probability of having a closer bus stop as the number of bus lines increases, we do not believe this affects their interpretation.

### 4.4. Strength and Limitations

This study has several strengths. A large and diverse sample of food aid recipients were surveyed at various locations, which allowed for multiple sub-analyses and nuanced examination of the understudied intersection of transportation and food aid. To our knowledge, it is the first study to assess this relationship with an integrated analysis of client- and panty-level characteristics. However, our study is not without limitations. First, this study was conducted as an in-person survey, which limits geographic reach and introduces inherent biases. Specifically, pantries and clients who declined or were unable to participate may differ systematically from those who participated. Language barriers may also have excluded those with limited English or Spanish proficiency. Additionally, our survey allowed participants to skip questions, leading to discrepancies in response rates. Second, the cross-sectional nature of the study limits its ability to draw conclusions on temporal or causal relationships. Relatedly, due to differences in pantry schedules and surveying resources, there was unavoidable variability in survey duration at each site. Each location also experienced varying levels of client traffic. Third, to our knowledge, no validated instruments exist that accurately capture our study goals. Thus, survey questions evaluating transportation methods and perceptions were developed. Further research is needed to assess their accuracy and reliability. Lastly, our statistical analysis was complicated by small sample sizes in stratified tests and multicollinearity in regressions, both of which may limit our ability to detect statistical significance and the interpretation of results. Concerns regarding multicollinearity were mitigated by either dropping highly collinear variables or conducting separate regression analyses.

## 5. Conclusions

This study finds significant associations between transportation resources and access to food aid, which contributes to the growing body of evidence that demonstrates transportation as a structural determinant of food security. We further elaborate on the interplay of client- and pantry-level characteristics in the context of food aid accessibility. Our findings are of particular concern given the increased food insecurity and compounding vulnerabilities seen in those who walk, bike, or take a bus to the food pantry, indicating that food aid is least accessible to those who need it the most. Transportation disadvantage seems most ameliorated by less restrictive pantry use policies and more robust public transit. Ensuring equitable access to food aid is a complex and dynamic issue. It demands coordinated, targeted interventions across multiple levels of government and aid organizations to efficiently allocate resources such that access to aid is not restricted for transportation-vulnerable populations. To further assess the causal relationship of these variables, future research should evaluate the longitudinal effects of changes in organizational policy and transportation infrastructure on access to aid. Together, these studies would drive contextually appropriate, community-informed strategies to improve equitable access to food resources.

## Figures and Tables

**Table 1:** Methods of Transportation: Most Frequent vs. To Pantry.

	Most Frequent Transportation		*p*-Value
	Walk/Bike/Bus (*n* = 120)	Car (*n* = 262)	
Transportation to Pantry			<0.001
Walk/Bike/Bus (*n* = 71)	66 (55)	5 (2)	
Car (*n* = 311)	54 (45)	257 (98)	

Note: Only compared patrons who indicated most frequent transportation and transportation to the pantry.

**Table 2:** Food Insecurity Scores by Transportation Type.

	Mean Score	Variance	*p*-Value
Transportation to Pantry			0.0603
Personal Vehicle (*n* = 151)	4.70	8.16	
Friend’s Vehicle (*n* = 71)	5.62	7.64	
Bicycle (*n* = 10)	6.30	6.68	
Bus (*n* = 8)	6.00	6.29	
Rideshare Service (*n* = 3)	6.67	2.33	
Walk (*n* = 24)	5.71	9.52	

**Table 3:** Transportation Perception Questions Based on Transportation Type.

	Ease of Transport Affects What Food I Select *		Transportation is Frequently Difficult for Me **		Transportation is Frequently a Financial Burden for Me ***	
	Large to Moderate Effect (*n* = 145)	Small to No Effect (*n* = 251)	Agree (*n* = 144)	Disagree (*n* = 134)	Agree (*n* = 201)	Disagree (*n* = 127)
Personal Vehicle	72 (50)	153 (61)	56 (39)	89 (66)	101 (50)	73 (57)
Friend’s Vehicle	36 (25)	65 (26)	52 (36)	26 (19)	60 (30)	30 (24)
Bicycle	12 (8)	6 (2)	10 (7)	3 (2)	11 (5)	5 (4)
Bus	9 (6)	9 (4)	7 (5)	4 (3)	9 (4)	6 (5)
Rideshare Service	2 (1)	1 (0)	3 (2)	0 (0)	3 (1)	0 (0)
Walk	14 (10)	17 (7)	16 (11)	12 (9)	17 (8)	13 (10)

Results reported as *n* (%). *p*-values: * = 0.0270, ** = 0.0002, *** = 0.4908.

**Table 4:** Demographics of Food Pantry Clients (*n* = 430).

Demographic Variable *	*n* (%)
Mean Age (SD) **	54.04 (14.73)
Did not answer	38 (9)
Education	
8th Grade or Less	18 (4)
Some High School	84 (20)
High School Graduate or GED	166 (39)
Some College or 2-Year Degree	122 (28)
4-Year College Graduate	23 (5)
Graduate or Professional Degree	14 (3)
Did not answer	3 (1)
Relationship Status	
Single, Never Married	138 (32)
Partnered/Committed Relationship	34 (8)
Married	89 (21)
Divorced	79 (18)
Widowed	45 (10)
Did not answer	45 (10)
Race/Ethnicity	
White	225 (52)
African American	90 (21)
Hispanic or Latino	42 (10)
Other	14 (3)
Two or More	18 (4)
Did not answer	41 (10)
Mean Household Size in Persons (SD)	3.20 (1.91)
Did not answer	123 (29)

* Results reported as *n* (%) unless otherwise indicated. ** SD = Standard Deviation.

**Table 5:** Demographics of Food Pantry Clients by Transportation Type.

	Walk/Bike/Bus	Car	*p*-Value
Demographic Variable *	*n* = 67 (17)	*n* = 325 (83)	
Mean Age	50.31	54.81	0.0097
	*n* = 70 (16)	*n* = 357 (84)	
Education			0.4649
8th Grade or Less	1 (1)	17 (5)	
Some High School	18 (26)	66 (18)	
High School Graduate or GED	25 (36)	141 (39)	
Some College or 2-Year Graduate	22 (31)	100 (28)	
4-Year College Graduate	3 (4)	20 (6)	
Graduate or Professional Degree	1 (1)	13 (4)	
	*n* = 68 (17)	*n* = 317 (82)	
Relationship Status			
Single, Never Married	38 (56)	100 (32)	<0.0001
Partnered/Committed Relationship	10 (15)	24 (8)	
Married	4 (6)	85 (27)	
Divorced	11 (16)	68 (21)	
Widowed	5 (7)	40 (13)	
Partnered vs. Single Status			
Partnered	14 (21)	109 (34)	<0.0001
Not Partnered	54 (79)	208 (66)	
	*n* = 68 (17)	*n* = 321 (83)	
Race/Ethnicity			0.0004
White	27 (40)	198 (62)	
African American	24 (35)	66 (21)	
Hispanic or Latino	5 (7)	37 (12)	
Other	6 (9)	8 (2)	
Two or More	6 (9)	12 (4)	

* Results reported using *n* (%) unless otherwise indicated.

**Table 6:** Mean Household Size (in Persons) by Transportation Type.

	Mean Household Size	Variance	*p*-Value
Transportation to Pantry			0.0065
Personal Vehicle (*n* = 181)	3.46	3.94	
Friend’s Vehicle (*n* = 83)	3.10	2.77	
Bicycle (*n* = 11)	2.27	2.02	
Bus (*n* = 10)	2.20	1.73	
Rideshare Service (*n* = 2)	2.00	0.00	
Walk (*n* = 20)	2.45	5.21	

**Table 7:** Multivariable Logistic Regression: Odds Ratios and *p*-values by Individual Characteristics.

Characteristic (*n* = 176)	Odds Ratio (95% CI)	*p*-Value
Age	1.02 (0.98–1.07)	0.371
Food Insecurity Score	1.28 (1.01–1.63)	0.043
Household Size, persons	0.53 (0.34–0.82)	0.004
Relationship Status		
Single, Never Married *	Ref	Ref
Partnered/Committed Relationship	0.31 (0.03–3.56)	0.349
Married	0.09 (0.01–0.88)	0.039
Divorced	0.18 (0.04–0.76)	0.020
Widowed	0.12 (0.01–1.37)	0.088
Race		
White *	Ref	Ref
African American	4.49 (1.21–16.72)	0.025
Hispanic or Latino	1.72 (0.17–17.50)	0.645
Other	10.14 (1.27–81.23)	0.029
Two or More	0.54 (0.05–6.01)	0.617

* The most common response among clients was utilized as a reference standard within each characteristic domain.

**Table 8:** Pantry Operation Characteristics by Transportation Type.

	Method of Transportation			*p*-Value
	Total (*n* = 430)	Walk/Bike/Bus (*n* = 72)	Car (*n* = 358)	
Days of Operation				<0.001
≥3 days/week	212 (49)	59 (82)	153 (43)	
<3 days/week	218 (51)	13 (18)	205 (57)	
Use Limits				<0.001
>1/month	175 (41)	44 (61)	131 (37)	
1/month	255 (59)	28 (39)	227 (63)	

**Table 9:** Pantry Food and Distribution Characteristics by Transportation Type.

	Method of Transportation			*p*-Value
	Total (*n* = 430)	Walk/Bike/Bus (*n* = 72)	Car (*n* = 358)	
Provision of Ready-Made Meals				<0.001
Yes	200 (47)	54 (75)	146 (41)	
No	230 (53)	18 (25)	212 (59)	
Client Choice				0.692
Complete Choice	351 (82)	61 (85)	290 (81)	
Partial Choice	41 (9)	5 (7)	36 (10)	
No Choice	38 (9)	6 (8)	32 (9)	

**Table 10:** Pantry Transit and Pedestrian Infrastructure by Transportation Type.

	Method of Transportation			*p*-Value
	Total (*n* = 430)	Walk/Bike/Bus (*n* = 72)	Car (*n* = 358)	
Distance to Bus Stop				<0.001
<0.25	129	50	79	
0.25–0.50	143	20	123	
0.50+	158	2	156	
Bus Lines				<0.001
0	129 (30)	2 (3)	127 (35)	
1	83 (19)	11 (15)	72 (20)	
2–3	123 (29)	19 (26)	104 (29)	
3+	95 (22)	40 (56)	55 (15)	
Sidewalk				0.047
Present	401 (93)	71 (99)	330 (92)	
Absent	29 (7)	1 (1)	28 (8)	
Bike Lane				<0.001
Present	301 (70)	70 (97)	231 (65)	
Absent	129 (30)	2 (3)	127 (35)	

**Table 11:** (**a**). Multivariable Logistic Regression: Odds Ratios and *p*-values by Pantry Characteristics. (**b**). Multivariable Logistic Regression: Odds Ratios and *p*-values by Pantry Characteristics.

(**a**)		
**Characteristic (*n* = 430)**	**Odds Ratio (95% CI)**	***p*-Value**
Distance to Nearest Bus Line		
>0.5 mi	Ref	Ref
0.25–0.5	18.16 (3.03–350.37)	0.008
<0.25	157.13 (25.30–3104.44)	<0.001
Days of Operation		
<3 days per week	Ref	Ref
3 or more days	0.50 (0.20–1.28)	0.142
Use Limits		
>1 visit per month	Ref	Ref
1 visit per month	0.18 (0.08–0.39)	<0.001
Sidewalks Present		
No	Ref	Ref
Yes	0.11 (0.00–2.95)	0.130
(**b**)		
**Characteristic (*n* = 430)**	**Odds Ratio (95% CI)**	***p*-Value**
Number of Bus Lines		
>3 Bus Lines	Ref	Ref
2–3 Bus Line	0.20 (0.10–0.42)	<0.001
1 Bus Lines	0.15 (0.05–0.40)	<0.001
0 Bus Lines	0.02 (0.00–0.10)	<0.001
Days of Operation		
<3 days per week	Ref	Ref
3 or more days	0.81 (0.31–2.14)	0.661
Use Limits		
>1 visit per month	Ref	Ref
1 visit per month	0.47 (0.24–0.93)	0.032
Sidewalks Present		
No	Ref	Ref
Yes	0.28 (0.01–7.30)	0.377

## Data Availability

The data presented in this study are available on reasonable request from the corresponding author due to ethical restrictions.
